# Immunological Basis of Bone Marrow Failure after Allogeneic Hematopoietic Stem Cell Transplantation

**DOI:** 10.3389/fimmu.2016.00362

**Published:** 2016-09-16

**Authors:** Stavroula Masouridi-Levrat, Federico Simonetta, Yves Chalandon

**Affiliations:** ^1^Division of Hematology, Department of Medical Specialties, Faculty of Medicine, Geneva University Hospitals, University of Geneva, Geneva, Switzerland

**Keywords:** bone marrow failure, graft failure, poor graft function, HSCT

## Abstract

Bone marrow failure (BMF) syndromes are severe complications of allogeneic hematopoietic stem cell transplantation (allo-HSCT). In this paper, we distinguish two different entities, the graft failure (GF) and the poor graft function (PGF), and we review the current understanding of the interactions between the immune and hematopoietic compartments in these conditions. We first discuss how GF occurs as the result of classical alloreactive immune responses mediated by residual host cellular and humoral immunity persisting after conditioning and prevented by host and donor regulatory T cells. We next summarize the current knowledge about the contribution of inflammatory mediators to the development of PGF. In situations of chronic inflammation complicating allo-HSCT, such as graft-versus-host disease or infections, PGF seems to be essentially the result of a sustained impairment of hematopoietic stem cells (HSC) self-renewal and proliferation caused by inflammatory mediators, such as interferon-γ (IFN-γ) and tumor necrosis factor-α, and of induction of apoptosis through the Fas/Fas ligand pathway. Interestingly, the production of inflammatory molecules leads to a non-MHC restricted, bystander inhibition of hematopoiesis, therefore, representing a promising target for immunological interventions. Finally, we discuss immune-mediated impairment of bone marrow microenvironment as a potential mechanism hampering hematopoietic recovery. Better understanding of immunological mechanisms responsible for BMF syndromes after allo-HSCT may lead to the development of more efficient immunotherapeutic interventions.

## Introduction

Allogeneic hematopoietic stem cell transplantation (allo-HSCT) is standard of care for many hematological diseases, and sustained engraftment of donor stem cells represents a fundamental prerequisite for a good outcome. Bone marrow failure (BMF) syndromes remain severe complications of allo-HSCT associated with considerable morbidity and mortality, highlighting the need for therapeutic manipulation. In this paper, we review the current understanding of the complex interactions between the immune and hematopoietic compartments in BMF syndromes after allo-HSCT.

Two entities with different clinical and physiopathological features can be distinguished: the graft failure (GF) and the poor graft function (PGF). Unfortunately, in a large number of studies, there is not a clear distinction between GF and PGF. Other frequent limitations of the literature are the lack of separation between primary and secondary cases and the heterogeneity of definition criteria. Primary GF is characterized by the lack of donor engraftment and is defined as never having achieved absolute neutrophil count ≥0.5 × 10^9^/L lasting for at least three consecutive days without evidence of disease relapse (Table 1). Thus, in case of myeloablative conditioning, the patient never recovers from the neutropenia making second allo-HSCT the only therapeutic option; while in case of reduced intensity conditioning (RIC), an autologous recovery may occur. Secondary GF refers to the loss of a previously functioning graft (1) associated with loss of full donor chimerism. Therefore, in both primary and secondary GF, chimerism status is either mixed, with the presence of recipient cells, or full recipient. GF incidence ranges from 3.8 to 5.6% (1–3) and varies significantly according to different transplant settings. Factors associated with increased risk for GF are HLA-mismatched grafts, RIC regimens (4), bone marrow (BM) grafts, low stem cell dose, non-malignant disorders, major ABO incompatibility, female donor grafts for male recipients (1–3), myeloproliferative disease (5), and disease status at transplantation (3). In contrast to GF, PGF is characterized by the presence of a full donor chimerism status (Table 1) as donor engraftment is achieved and sustained (6). Graft function can be poor either as a result of incomplete hematological recovery (primary PGF) or of a decrease of blood counts after prompt recovery (secondary PGF) (7). It is diagnosed in patients with two or three cytopenic lineages, hypoplastic/aplastic BM and full donor chimerism (Table 1). PGF occurs in 5–27% of patients (7) and it is associated with several post allo-HSCT conditions, such as infections, mostly viral, use of myelotoxic drugs, and graft-versus-host disease (GvHD) (8). Of note, GvHD, a common post allo-HSCT complication with an overall incidence up to 59% (9), represents the major risk factor for developing PGF (10) either in the context of the recently identified entity of BM GvHD (11, 12), or more usually, without specifically targeting BM. Thus, cytopenias are observed in ~40% of GvHD patients (13), while PGF is also seen in the absence of GvHD.

**Table 1:** **Clinical characteristics of graft failure and poor graft function**.

		Initial donor engraftment	Initial hematologic recovery	Cytopenias	Relapse	Bone marrow	Chimerism status
Graft failure	Primary	No	No	Yes	No	Hypocellular	Mixed or full recipient
	Secondary	Yes	Yes				
Poor graft function	Primary	Yes	No	Yes	No	Hypocellular	Full donor
	Secondary	Yes	Yes				

## Immunological Basis of Graft Failure

**Figure 1:**
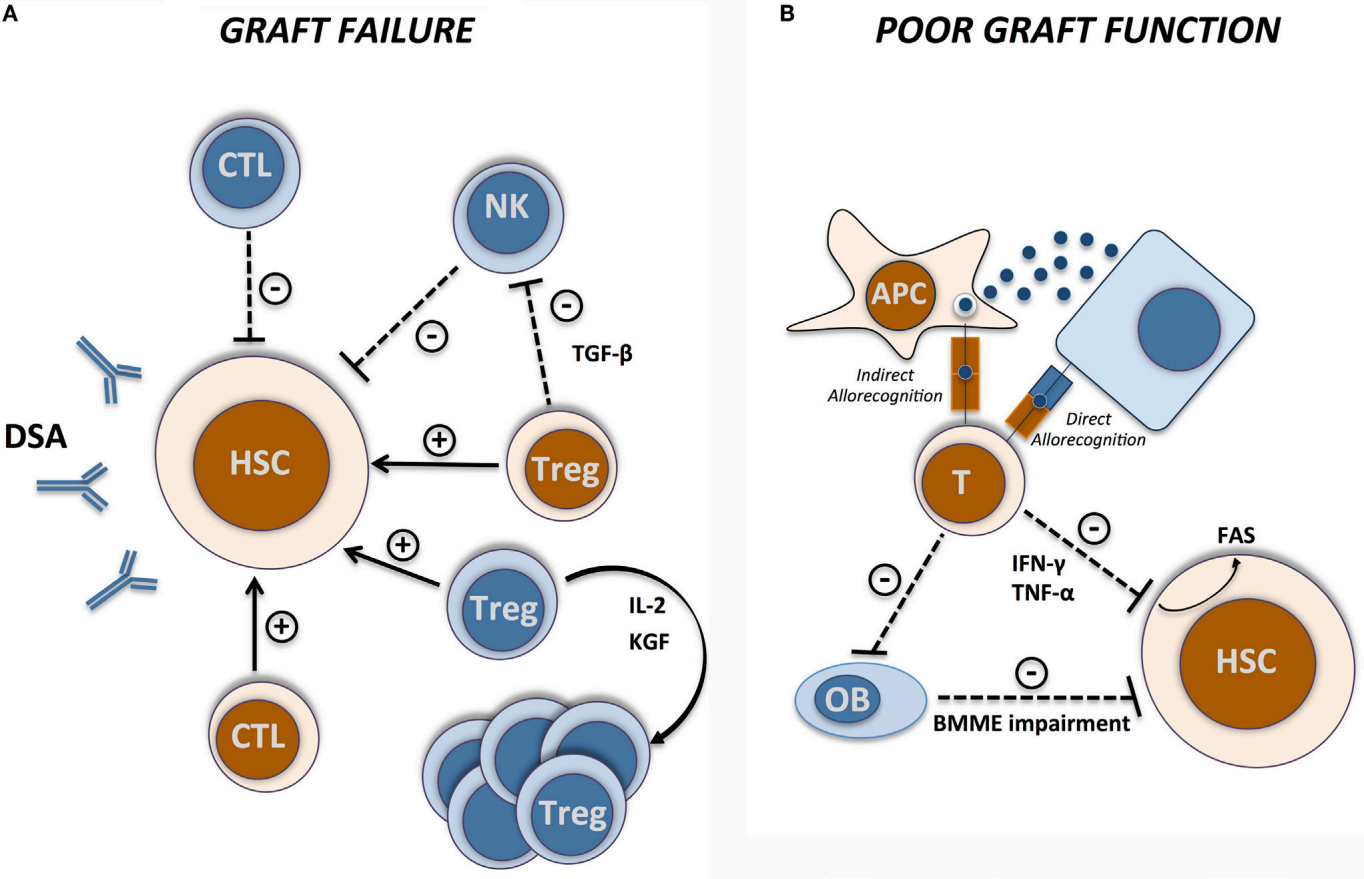
**Immunological basis of GF and PGF**. Immunological interactions between host (blue) and donors (brown) cells during **(A)** graft failure and **(B)** poor graft function. Arrows indicate facilitating effects and dashed lines indicate inhibitory effects. CTL, cytotoxic T lymphocyte; APC, antigen-presenting cell; OB, osteoblast; BMME, bone marrow microenvironment.

Graft failure occurs as the result of a classical alloreactive immune response mediated by residual host immunity persisting after the conditioning regimen (Figure 1A). Residual host T cells are considered the most prominent effector cells mediating rejection (14). Importantly, T-cell-mediated graft rejection can occur in both HLA-mismatched (15) and HLA-matched (16) settings, in the latter case as a result of responses directed against minor histocompatibility antigens (MiHA). The male-specific H-Y antigen represents a good example of a target MiHA involved in graft rejection (17, 18), increasing the risk of GF in sex mismatched transplantations (2, 19). The molecular pathways involved in T-cell-mediated graft rejection are still not completely defined and are probably multiple and somehow redundant as ablation of perforin, FasL, tumor necrosis factor receptor-1 (TNFR-1), and of tumor necrosis factor ligands TRAIL, TWEAK, and TL1A fails to prevent rejection (20, 21). Conversely, donor cytotoxic T cells are well known to mediate a facilitative effect on HSC engraftment (22–24) while graft T-cell depletion is associated with increased incidence of GF (25–27).

Residual host natural killer (NK) cells can also eliminate donor HSC as demonstrated in early studies in animal models (28–30). NK-mediated graft rejection, mainly observed in MHC-mismatched transplantations, is the result of a “missing-self recognition,” when the inhibitory receptors expressed on donor NK cells, belonging to the Ly49 family in mice and to the killer immunoglobulin-like receptors (KIRs) in humans, fail to recognize their cognate MHC class I molecule on host cells (31). Graft rejection by host NK cells is, at least partially, dependent on perforin-mediated cytotoxicity (32, 33). Similar to donor T cells, donor NK cells can facilitate HSC engraftment. In mice, adoptive transfer of donor NK cells facilitates HSC engraftment (34, 35), at least partly by abrogating the resistance of residual host effector immune cells to the graft. In humans, a similar effect has been observed in the HLA-haploidentical setting as transplantation with KIR ligand incompatible cells in the graft-versus-host direction conferred a significant protection from graft rejection in patients with acute myeloid leukemia (36).

The role of antibody-mediated rejection after allo-HSCT is much more controversial (37, 38). Recent studies demonstrate that the presence of donor-specific HLA antibodies (DSA), resulting in most cases from previous transfusion-induced sensitization, is associated with GF in HLA-mismatched (39–42) and haploidentical (42, 43) transplantations. Nevertheless, it is not clear whether DSA can actually mediate graft rejection or if they are surrogate markers for the cellular immunity that causes graft rejection. Recently published data suggest that integrated humoral and cellular immunity recognizing the same alloantigen of the donor can mediate graft rejection in DSA-positive patients undergoing HLA-mismatched cord blood allo-HSCT (44). Furthermore, patients displaying pretransplant donor-specific antibodies directed against CD34+/VEGFR-2+ endothelial progenitor stem cells presented a higher risk of GF, providing additional evidence supporting antibody-mediated rejection (45).

CD4^+^CD25^+^ regulatory T cells (Tregs) are critical immunomodulatory cells participating to immune and hematopoietic compartment interactions. In a syngeneic setting, Treg ablation enhanced early post-transplant hematopoiesis while co-administration of Tregs at time of transplantation inhibited myelopoiesis (46). Conversely, in the allogeneic setting, an engraftment facilitating effect of both host and donor Tregs seems to exist. Host Tregs prevent GF as their prior removal using anti-CD25 mAbs strongly enhanced allogeneic BM rejection in mice (47). Accordingly, adoptive transfer of host-type Tregs improved durable engraftment of allogeneic BM grafts (48–50). Interestingly, high resolution *in vivo* imaging showed marked co-localization of HSCs with host Tregs on the endosteal surface in the calvarial and trabecular BM, while after Treg depletion HSCs were lost, suggesting a direct effect of Tregs in HSC niches generation and maintenance (51). This effect seems to be essentially dependent on IL-10 production by Treg as IL-10 blockade by monoclonal antibodies or the use of Tregs isolated from IL-10 KO mice prevent the Treg-mediated protection of HSCs (51).

Donor Tregs seem as well to exert an engraftment facilitating effect without causing GvHD. In a fully MHC-mismatched BMT murine model, the co-transplantation of donor Tregs into sublethally conditioned recipients resulted in decreased early rejection of hematopoietic progenitors and improved long-term donor chimerism without inducing GvHD (52). The precise mechanisms through which host and donor Tregs exert their function on hematopoiesis are still incompletely understood. Transforming growth factor-β (TGF-β), a key mediator of Treg function, seems to play a role in Treg-mediated inhibition of IL-3-induced colony-forming units (46). Similarly, TGF-β is also involved in Treg engraftment facilitation as anti-TGF-β mAb treatment prior to allo-HSCT led to a significant increase in NK cell-mediated graft rejection, suggesting that Tregs mediate NK cell suppression through TGF-β (47). A major limitation of Treg-based therapies comes from the difficulties to isolate them from peripheral blood in sufficient amounts for adoptive transfer. Use of freshly isolated or *ex vivo* expanded third-party Tregs (53) or pharmacological approaches to induce Treg expansion *in vivo* represent potential alternatives. Administration of IL-2/anti-IL-2 mAb complexes to RIC conditioned mice early after MHC-matched allogeneic HSCT induces a strong expansion of host Tregs that efficiently facilitate early and long-term engraftment (54). Of note, in the absence of prior cytoreductive treatment, IL-2/anti-IL-2 complexes administration failed to promote BM engraftment as its effect was extended to several lymphocytes populations (55). In addition to IL-2, other molecules inducing *in vivo* expansion of Tregs display a potential to improve engraftment, including keratinocyte growth factor (KGF). KGF facilitated engraftment in an MHC-matched HSCT murine model by increasing the frequency of Tregs and enhancing their *in vivo* immunosuppression ability (56). Importantly KGF lost its ability to improve engraftment in Scurfy mice that lack Tregs.

Mesenchymal stem cells (MSCs) are stromal-derived multipotent progenitors displaying immune-modulatory properties of potential interest in HSCT [recently reviewed in Ref. (57)]. Several pilot studies performed in humans reported accelerated hematopoietic engraftment when MSCs were co-transplanted with HSC (58, 59), suggesting a potential for MSCs in preventing (58–61) and treating (58) GF.

## Immunological Basis of Poor Graft Function

While GF/rejection derives from “classical” alloreactive immune responses, PGF is the consequence of more complex and less well-defined interactions between the immune system and the hematopoietic compartment (Figure 1B). Important insights into the alloreactive immune responses involved in BMF syndromes first came from murine models of GvHD or aplastic anemia. Parental lymph node (LN) cells infusion into hybrid F1 with MHC-mismatch either sublethally irradiated (62) or not (63) induced a drastic and cell dose-dependent reduction of hematopoietic progenitors and stem cells. Marrow aplasia was associated with massive infiltration by T cells, mainly of the CD8^+^ compartment (62). The observation of strongly increased interferon-γ (IFN-γ) concentrations in these models (62, 63) pointed to IFN-γ produced by alloreactive T cells as the main effector molecule mediating BMF in this setting. Similar results were reported in a MHC class II-disparate, CD4-dependent model of GvHD in which BM aplasia was associated with massive infiltration by CD4^+^ T cells secreting IFN-γ (64). Interestingly, IFN-γ producing CD4^+^ T cells in this model expressed high levels of CXCR4 at their surface, suggesting a preferential BM homing potential. The relevance of IFN-γ as a key mediator of PGF in humans is supported by the observation of higher proportions of IFN-γ producing CD4^+^ and CD8^+^ T cells and decreased proportions of IL-4-producing T cells in BM from PGF patients, resulting in a shift of the IFN-γ/IL-4 ratio toward a type-1 immune response (65). IFN-γ and IL-4 levels in BM plasma were consistent with cellular results. The formal demonstration of the impact of IFN-γ in alloreactive PGF came from the observation that CD4^+^ T cells isolated from IFN-γKO mice adoptively co-transferred along with T-cell-depleted BM cells into sublethally irradiated recipients failed to induce the BM aplasia observed when WT CD4^+^ T cells were employed (66). Conversely, administration of IFN-γ receptor-1 deficient (IFN-γ-RKO) BM cells resulted in reduced severity of BMF (64). The use of IFN-γ-RKO recipients, in which extremely high, supraphysiological concentrations of IFN-γ are reached as a consequence of accumulation of IFN-γ produced by alloreactive T cells (67) or after inflammatory stimulation (68), provides complimentary information. In the MHC-matched, mHAg-mismatched C3H.SW > C57BL/6 transplantation model, donor HSCs were exposed to high levels of IFN-γ produced by alloreactive T cells leading to their decreased proliferation and finally to BM aplasia (67). A similar inhibition of HSCs was reported even when donor HSCs were transferred into syngenic IFN-γ-RKO mice in which extremely high levels of IFN-γ were induced through *Mycobacterium bovis* bacillus Calmette–Guérin (BCG) infection (68). Interestingly, the inhibitory effect of IFN-γ on hematopoiesis finally leads to lack of HSC engraftment despite the absence of alloreactive responses given the syngenic setting. Accordingly, using the already discussed BMF model induced by MHC-mismatch LN cells infusion, Chen and coworkers showed that co-transplantation of BM cells from BMF mice and from syngeneic healthy mice led to the destruction of hematopoietic progenitor stem and stromal cells from fully compatible healthy donors as “innocent bystanders” and not as targets (62). These observations suggest that, once elevated levels of IFN-γ are established, a non-HLA restricted inhibitory action is exerted on HSCs.

IFN-γ is a well-known negative regulator of HSC as demonstrated since earliest *in vitro* studies in both murine (69, 70) and human (71, 72) cells. IFN-γ seems to facilitate HSC programmed cell death either directly (73) or through induction of caspase-1, TRAIL (74), and FAS (75). Moreover, IFN-γ inhibits HSC self-renewal modulating expression of key cell-cycle genes, such as cyclin D1 (67, 76), Myc (67), and p57 (76). Notably, IFN-γ-mediated cyclin D1 suppression seems to essentially affect the immature LSK (Lin^−^Sca-1^+^c-kit^+^) hematopoietic stem/progenitor cell (HSPC) subset but not mature Lin^+^ cells (67). Finally, IFN-γ interferes with thrombopoietin-induced phosphorylation of signal transducer and activator of transcription-5 (STAT-5) in HSCs through modulation of suppressor of cytokine signaling (SOCS) 1 (76), further participating to inhibition of HSC proliferation through repression of STAT-5 target genes.

Collectively, these results suggest that in situations of chronic inflammation post allo-HSCT, such as GvHD or viral infections, PGF is, at least partially, the result of a sustained impairment of HSC self-renewal and proliferation caused by chronic IFN-γ exposure. In mice, IFN-γ blockade by administration of anti-IFN-γ neutralizing antibodies significantly improved cytopenias reversing or preventing BM aplasia in all aforementioned BMF models in which this strategy was tested (62, 63, 67, 68). IFN-γ, therefore, probably represents the most promising target for immunotherapeutic interventions in PGF. Targeted treatment with anti-IFN-γ blocking antibodies (NI-0501) is currently under clinical evaluation in primary hemophagocytic lymphohistiocytosis (NCT01818492), another setting of immune-mediated BMF.

Tumor necrosis factor-α (TNF-α) represents another inflammatory cytokine that, if overexpressed, may participate in post-allo-HSCT PGF mediation. Accumulating evidence has been established regarding the inhibitory role of TNF-α in clonogenic progenitor cells proliferation *in vitro* (77, 78). Incubation of LSK murine HSPC with TNF-α has been shown to upregulate Fas expression and to inhibit colony formation *in vitro* by reducing the size – but not the number – of proliferative clones (79), suggesting that HSCs can undergo a limited number of cell divisions before becoming sensitive to TNF-induced growth inhibition. Transplantation of these cells into lethally irradiated mice resulted in reduced short-term engraftment and long-term reconstituting activity, showing the impact of TNF-α on HSC function (79). Similarly, in a xenograft model of human CD34^+^CD38^−^ cells treated with TNF-α *in vitro* and transplanted into NOD–SCID mice, their repopulating ability was dramatically compromised. A negative effect of TNF-α on HSC maintenance by enhancing their differentiation rather than self-renewal has been suggested (80). However, there is no general agreement about the *in vivo* role of TNF-α in HSC regulation. In BMF murine models, treatment with anti-TNF-α mAbs resulted in significantly prolongation of animal survival although less impressive than using anti-IFN-γ mAbs treatment (63). Furthermore, using mice deficient for either Tnfrsf1a receptor or Tnfrf1b receptor or both, a TNF-mediated suppression of HSCs activity was shown (81). Of note, it was dependent on both receptors as *Tnfrsf1aKO* and *Tnfrsf1bKO* BM cells presented a greater ability to long-term reconstitute myeloid and lymphoid cell lineages in myeloablated WT recipients compared to WT BM cells. This advantage was more pronounced when *Tnfrsf1-doubleKO* BM cells were used (81). It was also shown that *in vivo* administration of TNF targets actively cycling rather than quiescent HSCs, finding of relevance in the early post-allo-HSCT setting where HSCs are recruited from quiescence to active proliferation. Conversely, a positive role of TNF-α in HSC engraftment and function has been reported in models of purified HSC transplantation into allogeneic and syngeneic mice in which small numbers of BM-derived CD8^+^/TCR^−^ cells have been shown to act as graft facilitating cells through a TNF-α-dependent mechanism (82).

In addition to soluble inflammatory mediators, substantial evidence implicates the Fas/Fas ligand pathway in the post-allo-HSCT PGF and mainly in the myelosuppressive effect of GvHD. Fas is expressed at CD34^+^ HSC surface (75, 83) and its expression is modulated by several factors, including IFN-γ and TNF-α (75). HSC exposure to agonist anti-Fas antibodies inhibits hematopoiesis by inducing HSC apoptosis (75, 83) and enhancing the effects of IFN-γ and TNF-α (75). *In vivo*, the Fas/FasL pathway has been shown to be relevant for BMF associated with cytomegalovirus infection (84) and GvHD (62, 63, 67, 83). In GvHD models, the use of FasL-defective (gld) donor cells prevented BM aplasia (83). Similarly, blockade of Fas/FasL interaction by the use of anti-FasL blocking antibodies significantly reduced cytopenias and BM aplasia (67, 83).

Hematopoiesis depends on special BM microenvironments known as “niches” in which HSCs reside as well as on the functional cross-talk between HSCs and these niches (85). BM endosteal, perivascular and vascular endothelial cells as well as osteoblasts have a fundamental role in the maintenance of HSCs by providing signals that regulate cell self-renewal, differentiation, and quiescence in mice (86). Two prospective nested case-control studies provided evidence that endosteal, perivascular cells, and endothelial progenitor cells are dramatically reduced in patients with primary and secondary PGF, suggesting that an impaired BM microenvironment hampers hematopoietic recovery after allo-HSCT (87, 88). The etiology of BM microenvironment impairment and how this leads to PGF remain unclear. Studies of BM GvHD in murine models identified osteoblasts as a major target for GvHD. Donor effector CD4^+^, and to a lesser extent CD8^+^, T cells caused early destruction of osteoblasts leading to severe impairment of B lymphopoiesis with dramatically decreased numbers of B-cell precursors and decreased expression of transcriptional factors essential for B lymphopoiesis, such as E2A and PAX5 (11). Surprisingly enough, there were no differences in serum levels of major inflammatory cytokines thought to be involved in GvHD, such as IL-1, IFN-γ, and TNF-α, suggesting that they are dispensable for CD4^+^ T cell-mediated osteoblast destruction. These findings were recently confirmed in allo-HSCT patients with PGF associated with chronic GvHD in which loss of osteoblasts, decreased numbers of B cells and an increased CD4/CD8 ratio were shown (89).

## Conclusion

Bone marrow failure syndromes, namely GF and PGF, can develop after allo-HSCT and are associated with significant morbidity and mortality. In the present paper, we summarized the distinct immune-pathological mechanisms underlying GF and PGF and we discussed evidence identifying several inflammatory molecules as crucial mediators in their pathogenesis. Some preclinical evidence obtained in murine studies suggest that therapeutic interventions blocking these inflammatory mediators may represent a promising strategy to prevent and even reverse these severe complications.

## Author Contributions

SM-L wrote the manuscript and designed the table and the figure. FS and YC critically discussed the work and edited the manuscript.

## Conflict of Interest Statement

The authors declare that the research was conducted in the absence of any commercial or financial relationships that could be construed as a potential conflict of interest.
